# Supplementary figures and images for: Infants’ brain activity to cartoon face using functional near-infrared spectroscopy
Source: PLoS One. 2022 Feb 16;17(2):e0262679. doi: 10.1371/journal.pone.0262679 (PMC8849497; doi:10.1371/journal.pone.0262679)

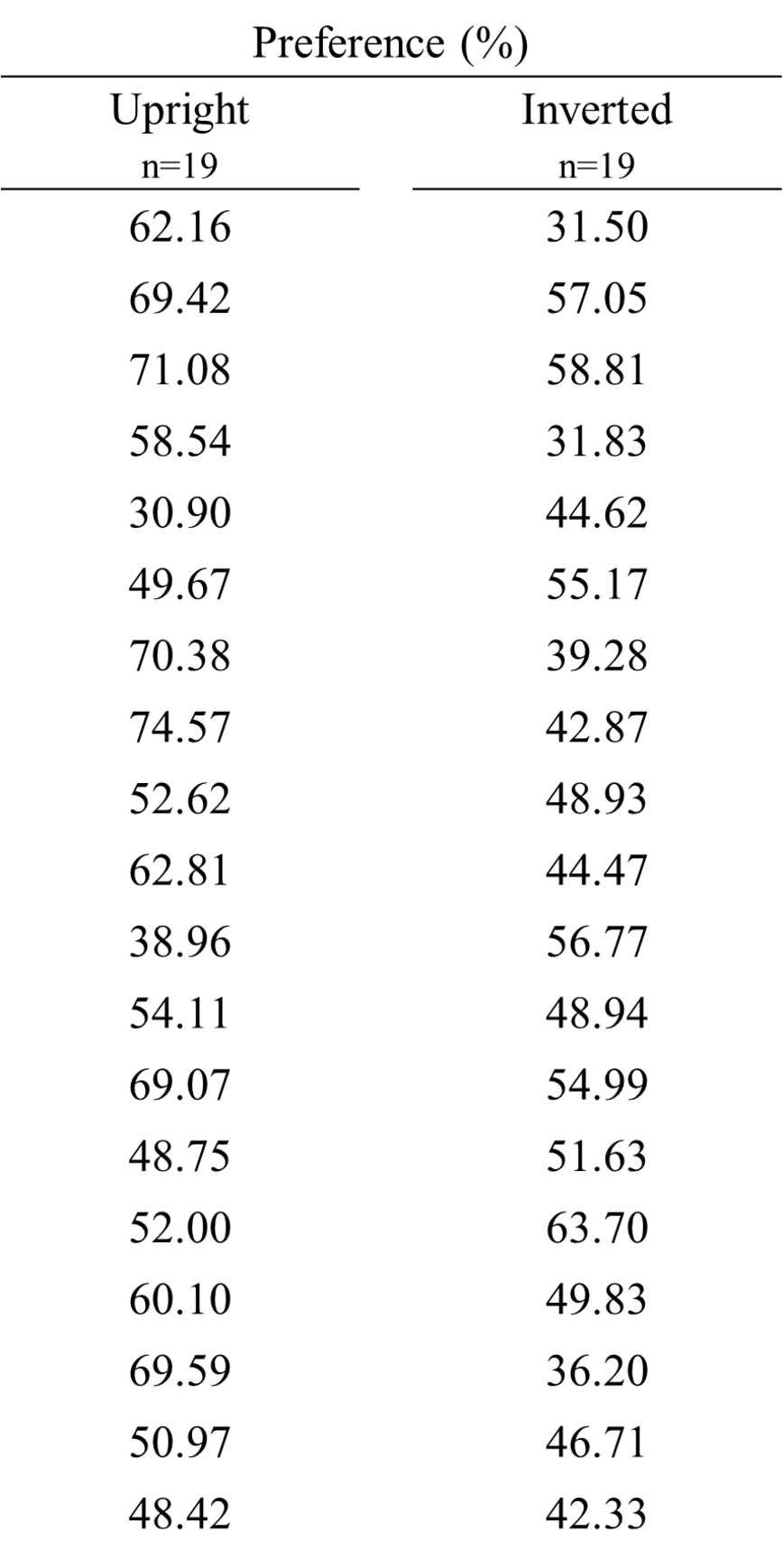

Supplement: S1 Table — (TIF) [file pone.0262679.s001.tif]

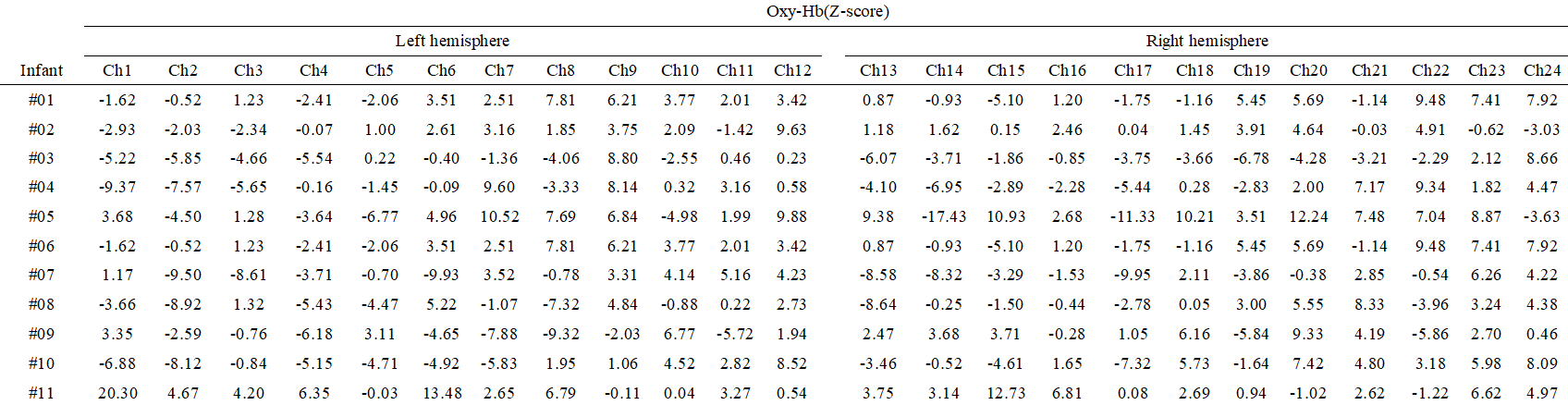

Supplement: S2 Table — (TIF) [file pone.0262679.s002.tif]

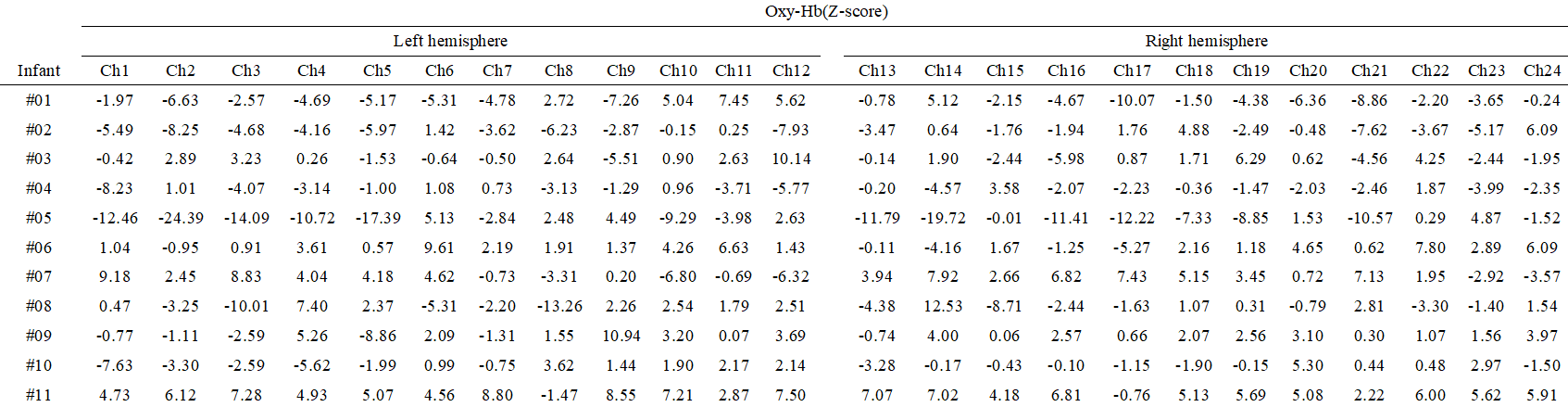

Supplement: S3 Table — (TIF) [file pone.0262679.s003.tif]

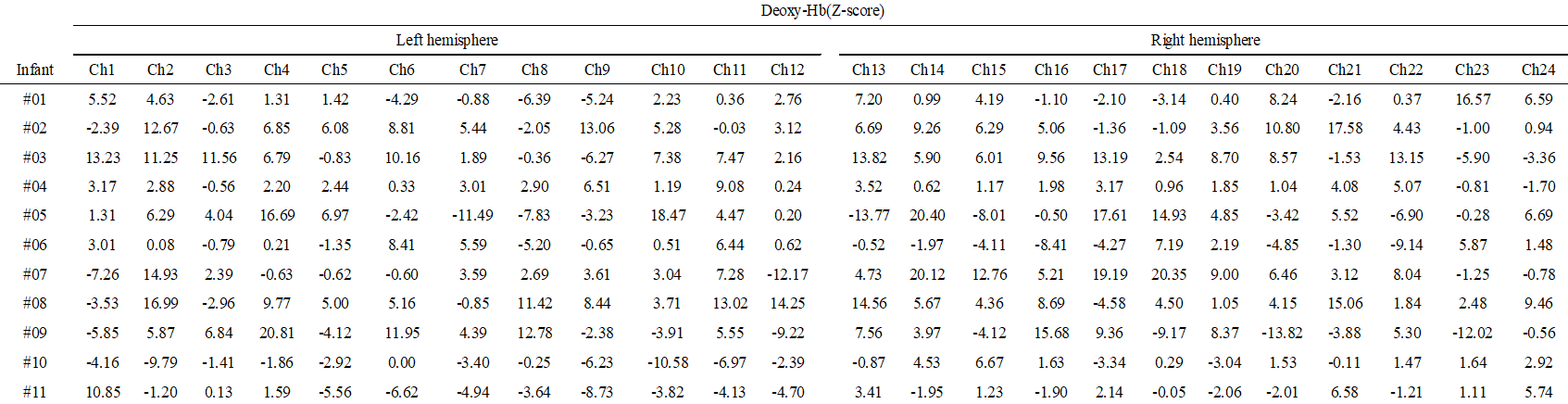

Supplement: S4 Table — (TIF) [file pone.0262679.s004.tif]

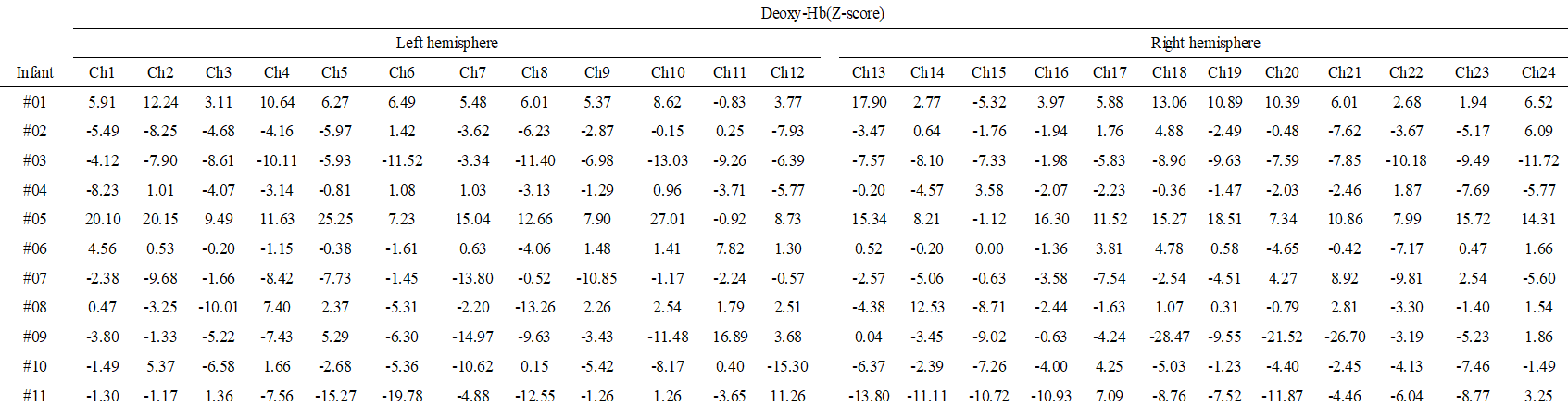

Supplement: S5 Table — (TIF) [file pone.0262679.s005.tif]
