# Supplementary material for: Infants’ brain activity to cartoon face using functional near-infrared spectroscopy
Source: PLoS One. 2022 Feb 16;17(2):e0262679. doi: 10.1371/journal.pone.0262679 (PMC8849497; doi:10.1371/journal.pone.0262679)
Supplement: Attachment — Submitted filename: Ref01.docx [file pone.0262679.s006.docx]

## Review Report

The authors investigated two face-stimuli-related experiments for 5- and 6-month-old infants. The behavioral one showed a preference for cartoon female faces in the upright, while another NIRS one showed that the concentration of oxy-Hb increased in the case of cartoon female faces. These results are, in general, interesting for readers. Some comments and suggestions should be considered in a modified version of the manuscript.

1. For any subject, the authors should obtain both behavioral measure data and NIRS imaging data simultaneously. Then, they should detect if there is correlative between behavioral and NIRS measurement.
2. The sample size is relatively small and there is a significant gender difference of sample. The authors should increase more samples in their study if possible.
3. The explanation of their results should be strengthened. More importantly, the mechanism underlying the current study should be discussed more deeply. In addition, it is expected to know if the current results can also be extended to infants with high risk of developmental disorder.
