# Supplementary material for: Infants’ brain activity to cartoon face using functional near-infrared spectroscopy
Source: PLoS One. 2022 Feb 16;17(2):e0262679. doi: 10.1371/journal.pone.0262679 (PMC8849497; doi:10.1371/journal.pone.0262679)
Supplement: Attachment — Submitted filename: Response to Reviewer.docx [file pone.0262679.s007.docx]

**Editor:**

**Journal Requirements:**

1. *Please ensure that your manuscript meets PLOS ONE's style requirements, including those for file naming.*

Response: We revised our manuscript to meet PLOS ONE’s style requirements.

2. *We note that the grant information you provided in the ‘Funding Information’ and ‘Financial Disclosure’ sections do not match.*

*When you resubmit, please ensure that you provide the correct grant numbers for the awards you received for your study in the ‘Funding Information’ section.*

Response: We ensured the correct grant numbers in the ‘Funding Information’ section.

3. *Thank you for stating the following in the Acknowledgments Section of your manuscript:* *This research was financially supported by “Construction of the Face-Body Studies in Transcultural Conditions” (17H06343).*

*Please note that funding information should not appear in the Acknowledgments section or other areas of your manuscript. We will only publish funding information present in the Funding Statement section of the online submission form.*

*Please remove any funding-related text from the manuscript and let us know how you would like to update your Funding Statement. Currently, your Funding Statement reads as follows:*

*This research was financially supported by “Construction of the Face-Body Studies in Transcultural Conditions” (17H06343).*

Response: We removed the funding-related text from our manuscript.

Our Funding Statement:

*Construction of the Face-Body Studies in Transcultural Conditions　(17H06343)*

4. *PLOS requires an ORCID iD for the corresponding author in Editorial Manager on papers submitted after December 6th, 2016. Please ensure that you have an ORCID iD and that it is validated in Editorial Manager. To do this, go to ‘Update my Information’ (in the upper left-hand corner of the main menu), and click on the Fetch/Validate link next to the ORCID field. This will take you to the ORCID site and allow you to create a new iD or authenticate a pre-existing iD in Editorial Manager.*

Response:　I created my ORCID iD and ensured that it is validated in Editorial Manager.

5. *We note that Figure 1 in your submission contain copyrighted images. All PLOS content is published under the Creative Commons Attribution License (CC BY 4.0), which means that the manuscript, images, and Supporting Information files will be freely available online, and any third party is permitted to access, download, copy, distribute, and use these materials in any way, even commercially, with proper attribution.*

Response: We supplied the replacement figures that complies with the CC BY 4.0 license and updated the figure caption (Fig.1(page 6, line 123 - 124) and Fig.3 (page 10, line 219 - 221)).

6. *We noticed you have some minor occurrence of overlapping text with the following previous publication(s), which needs to be addressed: https://www.sciencedirect.com/science/article/pii/S1053811918321098?via%3Dihub*

*https://www.sciencedirect.com/science/article/abs/pii/S0022096511001858?via%3Dihub*

*The text that needs to be addressed involves the Result, Data Analysis, and Introduction sections.*

*In your revision ensure you cite all your sources (including your own works), and quote or rephrase any duplicated text outside the methods section. Further consideration is dependent on these concerns being addressed.*

Response: We revised the overlapping text with the previous publications.

**Comments to the Author:**

4. *Is the manuscript presented in an intelligible fashion and written in standard English?*

*PLOS ONE does not copyedit accepted manuscripts, so the language in submitted articles must be clear, correct, and unambiguous. Any typographical or grammatical errors should be corrected at revision, so please note any specific errors here.*

*Reviewer #1: Yes*

*Reviewer #2: No*

Response: We had the entire manuscript proofread by a native English-speaking editor.

*5. Review Comments to the Author*

**Reviewer #1:**

*A novel study presented in this manuscript however, many details are unclear, and the manuscript needs improvement. The results presented are interesting however, the sample size is very small.*

Response: Thank you for your suggestion. As for the sample size, for behavioral study (Experiment 1), we obtained the additional data (page 5, lines 103 - 104). For fNIRS study (Experiment 2), we added the explanation about the sample size to Methods section (page 9, lines 183 - 184).

As for the other points that the reviewer pointed out, we revised our manuscript, as shown in the followings.

(page 5, lines 103 - 104)

38 healthy 5- and 6-month-old infants (17 boys and 21 girls, mean age of 164.71 days, range from 136-193 days) participated in the experiments.

(page 9, lines 183 - 184)

Sample size was based on previous studies testing infants’ cortical hemodynamic changes during observation of face stimuli [8, 13-14].

*Data analysis, Line 273:*

*It’s unclear what the authors mean by “raw oxy-Hb signal”. Where the changes in concentration calculated from the attenuation change data? If so, how was this done? Were in-house scripts used? What was the procedure? The use of just a band-pass filter seems highly minimal – did the authors not use any type of motion correction for the data as infant data can have many motion artifacts?*

Response: The NIRS system that we used in the current study, Hitachi ETG-4000, calculated the changes in concentration from the attenuation change data by using modified Lambert Beer law. We used the data of changes in concentration for data analysis, then calculated Z-score based on the data of changes in oxy-Hb and deoxy-Hb concentration. That is, “raw data” in the previous manuscript meant “changes in concentration.” To clarify this point, we revised the Data analysis section.

As for the motion correction, we excluded trials that observed sharp changes in the time series of oxy-Hb concentration. And we used just the band-pass filter to remove some artifacts (e.g., longitudinal signal drift and high frequency noise due to heartbeat pulsations). The procedures for motion correction in the current study is the same as those in the previous fNIRS studies testing infant’s brain activity in response to the face (Kobayashi et al., 2012; Nakato et al., 2009; Otsuka et al., 2007).

*Could the authors also report the deoxy-Hb signal results? It is important to look at both the changes in oxy-Hb and deoxy-Hb.*

Response: Based on your suggestion, we added the result of the change in deoxy-Hb (page 15, lines 322 - 325; page 16, lines 333 - 335).

(page 15, lines 322 - 325)

There were no channels showing significant changes in the concentration of deoxy-Hb (all *ps*> .10 after Bonferroni’s corrections). In the cartoon character face condition, there were no channels showing significant changes in the concentrations of oxy-Hb and deoxy-Hb (all *ps* > .10 after Bonferroni's corrections).

(page 16, lines 333 - 335)

There were no significant differences in deoxy-Hb concentration between presentation of cartoon female and cartoon character face condition in channel 23 (*t* (10) = 1.08, *p* > .10).

*The authors need to improve the data analysis section in Experiment 2 to reflect the points above and the deoxy-Hb results need to be added in. As it stands, the data analysis section is confusing and it’s unclear what exactly is being shown. It’s also unclear why the authors chose to present “z-score” of the “raw signal” rather than changes in concentration.*

Response: We wrote the reason for using “Z-score” (page 13, lines 285 - 287).

(page 13, lines 285 - 287)

The concentration changes measured by fNIRS were originally relative values and could not be averaged directly across participants. Normalized data such as the Z-scores, however, could be averaged regardless of the unit [20-21].

*Result, Line 315:*

*I believe that the statistical testing could be performed in a more accurate way. For example, rather than testing against 0, the mean z-score should be tested against the mean z-score in the baseline period. This would give a more accurate statistical comparison. Could the authors please repeat the statistical analysis?*

Response: First, we calculated Z-scored at each time point during the cartoon female face and cartoon character face condition for each channel within each participant. The Z-score(*d*) was calculated by subtracting the mean concentration of the baseline period (*m_baseline_*) from the concentration at each time point during the test period (*m_test_*), and dividing this difference by the standard deviation of the concentration data during the baseline period (*sd*), as follows:

*d* = (*m_test_* －*m_baseline_*) / *sd*

The 1 s immediately before the onset of each test trial was defined as the baseline.

Second, to examine whether the changes in hemoglobin concentration in the test period significantly increased or decreased compared to the baseline activation, two-tailed one sample t-tests versus a chance level of 0 (baseline period) were conducted for the mean Z-score of oxy-Hb and deoxy-Hb during the test period for all 24 channels.

As described above, we calculated the Z-scores in each test trial by using the mean concentration and the standard deviation of the last 1 s of baseline period immediately before the onset of each test stimulus presentation. Consequently, mean value during the baseline period was changed into Z-score 0, and standard deviation into Z-score 1 in all channels. That is, testing Z-scores against 0 means testing them against the mean Z-score in the baseline period. The statistical analysis in the current study is the same as those in the previous studies (e.g., Kobayashi et al., 2012; Nakato et al., 2009; Otsuka et al., 2007; Tsurumi et al., 2019).

To clarify the statistical analysis, we revised the data analysis section (page 14, lines 288 - 294) and discussion (page 17, lines 355 - 357) in the revised manuscript.

(page 14, lines 288 - 294)

Statistical analyses were conducted on these mean Z-scores for the oxy- and deoxy-Hb concentrations. To examine the channel showed greater activation for the cartoon female and cartoon character face comparing to the object (baseline), two-tailed one-sample t-tests vs. a chance level of 0 (baseline) were conducted for the mean Z-score in each of the 24 channels. Furthermore, to investigate the difference between presentation of the cartoon female and cartoon character face condition, we conducted two-tailed paired t-tests on the mean Z-scores.

(page 17, lines 355 - 357)

The result of the fNIRS experiment (Experiment 2) revealed that the 5- and 6-month-old infants showed increased concentration of oxy-Hb for cartoon female faces but not for cartoon character faces in channel 23.

*Discussion*

*The work presented in this paper seems novel in that this specific effect has not been studied in infants before this study. However, besides restating the results, the authors don’t offer any discussion or explanation of their results. For example, Channel 23 was the only channel that showed significant activation – do the authors have a hypothesis about this? Why is this channel specifically important for these stimuli? Most of the discussion section reads as a literature review with lots of studies cited. The authors need to work on the discussion and weave in the studies to support their hypotheses and results as opposed to how it is currently written.*

Response: Thank you very much for your suggestion. Per your suggestion, we added the discussion about the channel 23 (page 19, lines 407 - 419).

(page 19, lines 407 - 419)

In our study, channel 23 could discriminate between the cartoon female and cartoon character face. In other words, the 5- and 6-month-old infants showed an increase in oxy-Hb concentration for the cartoon female face but not for the cartoon character face in channel 23.　Previous fNIRS studies have also been suggested that channel 23 is activated for faces [9,17].　Nakato et al. found that channel 23 showed an increase in oxy-Hb concentration for profile-view face presentations in 8-month-old infants [9]. Kobayashi et al. found that channel 23 showed a reduction in the concentration of oxy-Hb for the adaptation of identical faces in 5- to 6-month-old infants [17]. These studies revealed that channel 23 was involved in the perception of profile view and the representation of facial identity. In our study, we found that channel 23 showed an increase in oxy-Hb concentration for the cartoon female face. Our study indicates that the cartoon female face, but not the photographed female face, could induce activity in channel 23.

**Reviewer #2:**

*1. For any subject, the authors should obtain both behavioral measure data and NIRS imaging data simultaneously. Then, they should detect if there is correlative between behavioral and NIRS measurement.*

Response: We think that we can’t conduct both fNIRS and behavioral experiment simultaneously for one participant. It takes about 30 minutes to 1 hour to conduct only fNIRS experiments. If infants participate to both behavioral and fNIRS experiments simultaneously, infants are likely to get fussy and bored, then it becomes difficult to conduct experiments. Therefore, to avoid this situation, we collected the behavioral and fNIRS data using a between-participants design. The previous studies also reported behavioral and fNIRS data that obtained from different participant groups (Kobayashi et al., 2012; Tsurumi et al., 2019).

*2. The sample size is relatively small and there is a significant gender difference of sample. The authors should increase more samples in their study if possible.*

Response: Per your suggestion, we obtained additional data to reduce a gender difference of sample. In experiment 1, the data of 4 boys is added in each of conditions (upright and inverted) (page 5, lines 103 - 104).

*3. The explanation of their results should be strengthened. More importantly, the mechanism underlying the current study should be discussed more deeply. In addition, it is expected to know if the current results can also be extended to infants with high risk of developmental disorder.*

Response: Thank you very much for your suggestion. We revised the Discussion section and added the following sentences about the future study in infants with high risk of autism (page 18, lines 394 - 406).

(page 18, lines 394 - 406)

Grelotti et al. investigated brain activity in response to the human face and Digimon in two boys with autism using fMRI [6]. In an 11-year-old boy who had a special interest in Digimon, brain activity in his fusiform gyrus was induced by Digimon but not the human face. In a 17-year-old boy who did not have a special interest in Digimon, brain activity in his fusiform gyrus was not induced by either Digimon or the human face. According to their study, the human face could not induce activity of the FFA in either an 11-year-old or a 17-year-old boy with autism. The current study shows that the occipito-temporal regions of the 5- to 6-month-old infants activated for the cartoon female face, but not for the cartoon character face. To summarize the two studies, the human face could induce activity in typically developing infants, but not in juveniles with autism. Future studies could investigate abnormal brain activity in response to the human face in 5- to 6-month-old infants at risk due to being younger siblings of children with autism. This task may aid in the diagnosis of autism in the early infant period.
